# Long-term changes in melanoma epidemiology in Hungary: who are the high-risk groups, and where should we concentrate prevention programs?

**DOI:** 10.1186/s12889-026-26713-w

**Published:** 2026-02-21

**Authors:** Ágnes Stier, István Kenessey, Anna Páldy

**Affiliations:** 1 Semmelweis University Doctoral College, Budapest, Hungary; 2 Hungarian National Cancer Registry, National Institute of Oncology, Budapest, Hungary; 3 National Center for Public Health and Pharmacy, Budapest, Hungary

**Keywords:** Melanoma, Incidence, Mortality, Hungary, Trend analysis

## Abstract

**Background:**

Numerous studies have concentrated on the epidemiology of melanoma in Hungary in recent years. The incidence rates often conflict in these studies; however, the prognosis has unambiguously improved. Our objective was to investigate melanoma epidemiology from a spatial perspective by relying on healthcare databases to identify high-risk groups and reveal geographical differences over a 20-year study period.

**Methods:**

We calculated age-standardised incidence rates in each Nomenclature of Territorial Statistical Level 3 (NUTS-3) region (county) for 2001–2019 for the 45–64 and 65 years or older populations, stratified by sex. We applied the European Revised Reference Population (2012) for standardisation. Furthermore, we used the Joinpoint Trend Analysis software to calculate the annual percent change (APC) to determine whether the incidence trends were statistically significant. We then analysed 10-year-specific APCs in the population above 45 years of age. Additionally, we performed the Joinpoint Trend Analysis on the age-adjusted death rates in the population between 45 and 64 years and 65 years or older at the NUTS-2 level.

**Results:**

Melanoma showed a 1.8% and 1.5% increase in melanoma incidence in individuals aged 45–64 years. ASIRs increased in men from 2010 to 2015 by 8.2% and in women without breakpoints by 4.1% between 2001 and 2019. We also demonstrated geographical differences in the incidence trends. We observed increasing trends in incidence rates in men in counties with low socioeconomic status, such as northern counties, and women in Southern Transdanubia. Our findings warrant further research on the relationship between melanoma incidence and socioeconomic status. Decreasing incidence trends from 45 to 64 years of age were observed in Vas and Hajdú-Bihar counties. Our most noteworthy finding pertains to the vulnerability of 45–54 year old women and highlights the urgency of efficient prevention and screening campaigns. Melanoma mortality in most regions stagnated or decreased, except for Western Transdanubia among the population above 65 years of age.

**Conclusion:**

Our study results could aid policymakers in designing awareness and screening campaigns in areas with a dynamically increasing incidence of melanoma. Particular attention should be given to areas with low socioeconomic status.

**Supplementary Information:**

The online version contains supplementary material available at 10.1186/s12889-026-26713-w.

## Background

Over the last four decades, the global incidence of melanoma has increased sixfold [1]. According to the estimates of GLOBOCAN, in 2022 more than 330 000 new cases were discovered, which means that this is the 17th most common malignant tumour type [2]. Arnold et al. (2022) reported that Australia, New Zealand, and Western and Northern Europe still share the majority of the melanoma burden. Central and Eastern Europe (CEE) had a middle rank in incidence, yet worldwide, the highest number of deaths occurred in 2020 [3]. Since the Central European region had the fifth highest cancer-related disability-adjusted life years (DALY), our current study has focused on Hungary within this region [4].

Melanoma incidence has been increasingly studied in Hungary. Several reports documented increasing age-standardised incidence rates [5–7], while at least one study showed that incidence rates shifted between 2011 and 2019 in Hungary, with rates declining for both males and females between 2015 and 2019 [8]. The explanation may be partly contradictory because these works differed in their study periods, data sources and statistical methods.

Melanoma mortality has declined in several high-income nations in recent decades due to advances in early diagnosis and efficient therapies. A decline was confirmed in the age groups 20–44 years and 45–64 years by De Pinto et al. [9], who analysed death certificates of selected WHO countries [9]. Koczkodaj et al. [10] reported that in Hungary the melanoma mortality rate decreased between 1960 and 2020 in both sexes in the 45 to 75-year-old age range; however, in the 75 year old or older population, the mortality rate increased in men [10]. Parrag et al. [6] reported a stagnation in mortality rates in Hungary between 2001 and 2019 for both sexes. They also found that the mortality-to-incidence ratio (M/I), widely used as a proxy for cancer survival, decreased [6]. Another study by Várnai et al. [11] investigated the M/I ratio between 2011 and 2018 and revealed that melanoma survival improved in both men and women [11]. Moreover, Liszkay et al. [12] confirmed an improved survival between 2011 and 2019 [12].

It is essential to understand how reporting practices influence melanoma incidence trends and how patient data are recorded in the database. In Hungary, the healthcare system follows a hybrid model, and people can receive a melanoma diagnosis through either public or private providers [13]. Dermatological care is available without referral to all patients with valid social security numbers [14]. After a preliminary diagnosis, the patient is referred to the oncodermatology unit for treatment. Chemo- and radiotherapy are provided by the public healthcare system [15]. Healthcare providers, both public and private have to report incident melanoma cases to the Hungarian National Cancer Registry (HNCR), which has been operated by the National Institute of Oncology since 2000, hence, we limited our study to a period after 2000 [16]. The efficiency of reporting by private healthcare providers remains unknown.

The publicly available HNCR database is limited to the Nomenclature of Territorial Statistical (NUTS) level 3 units. The EU established the NUTS for statistical and policy purposes: NUTS-1 represents major socio-economic regions, NUTS-2 is created for regional policies, and NUTS-3 refers to smaller administrative regions [17]. The NUTS levels in Hungary encompass 3 NUTS-1 regions, 8 NUTS-2 regions and 20 NUTS-3 regions (counties) [18]. Out of the 20 counties, Budapest and Pest account for 30% of the entire population and have better access to oncotherapy [19]. In Hungary, outpatient clinical oncology care is provided by 94 institutions, inpatient care by 88 institutions, and inpatient hospital care by 30 institutions. Additionally, 12 offer radiotherapy [20]. Individual patient data are kept by the National Health Insurance Fund (NHIF) [21]. Cause-specific mortality data is collected by the Hungarian Central Statistical Office (HCSO) [22].

Despite the growing literature on melanoma epidemiology in Hungary, we decided to investigate melanoma epidemiology from a spatial perspective, relying on health databases, namely the HNCR and the HCSO, to identify high-risk groups and regions. Additionally, our goal was to comprehend incidence trends at the NUTS-3 (county) level to prepare for a long-term district-level investigation of melanoma mortality and socioeconomic determinants.

## Methods

Data of cutaneous melanoma cases were obtained from the publicly available database of the population-based HNCR (available at: https://stat.nrr.hu/), based on recorded C43 code according to International Statistical Classification of Diseases and Related Health Problems 10th Revision (ICD 10) [23]. The query focused on the numbers of newly discovered cases between 2001 and 2019, categorised by sex, 5-year age groups and counties.

Age-adjusted incidence rates (ASIRs) per 100 000 people in the NUTS-3 region (20 counties) were calculated. The European Revised Reference Population (2012) served as reference [24]. HCSO supplied the population data (available at www.ksh.hu). In addition, this paper was prepared via a tabular data set for 5-year age intervals by sex compiled by the Central Statistical Office in response to a specific request [25].

We calculated the ASIR for two subcohorts, the 45–64 year-old population and the 65 year-old or older population, stratified by sex. We excluded the population younger than 45 years because the case numbers were negligible.

To study trends in the younger subgroup, we then analysed age-standardised rates in 10-year age bands (45–54, 55–64, 65–74 and 75–84).

Aggregated mortality data of melanoma between 2001 and 2019 were obtained from HCSO through an official data-sharing agreement between the National Center for Public Health and Pharmacy (NCPHP) and the HCSO. We calculated the region-specific age-adjusted death rate (ASDR) per 100 000 people for the populations aged 45–64 years and 65 years or older. Owing to the low case numbers at the county level, we kept the ASDR unstratified and chose the NUTS-2 region (8 regions) as the geographical unit of analysis.

We used the freely available Joinpoint Trend Analysis software (version 5.0.2; National Cancer Institute; available at https://surveillance.cancer.gov/joinpoint/*)* to analyse cancer trends. The analysis aims to detect the so-called joinpoints, which are breakpoints observed in the trends. The software allowed selection of the best-fit logarithmic linear model to estimate the annual percentage change (APC) and its 95% confidence intervals (CIs). A maximum of three joinpoints was allowed in the models. Only significant changes (CIs excluding zero) were considered for interpretation to describe trends. Interpretations of short time periods were excluded due to low case counts and small inter-annual variability. These estimates do not appear in the text but are included in the tables.

Ethical approval was waived for this study. HNCR is a publicly accessible database, and only aggregated data can be retrieved. The cause-specific mortality data obtained from HCSO is not publicly available. Access to these data is granted through official data sharing agreement between the NCPHP and the HCSO to support the official activities of the NCPHP, and secondary analyses of HCSO data is therefore needed to perform the statutory tasks of NCPHP. Furthermore, it is important to note that the two databases have not been linked. The NCPHP receives only aggregated data from the HCSO. Furthermore, HNCR also reports aggregated data of cases by year of diagnosis at NUTS-3 level, and HCSO publishes mortality data by year of death also at NUTS-3 level. The population size of the counties also precludes any possibility of cross-referencing individuals. Additionally, the county of diagnosis may differ from the county of death, further preventing any potential linkage.

## Results

The APCs for melanoma incidence are presented in Table 1, and 2. The crude and age-standardised rates are listed in Table 1, and 2 of the Supplementary Material. For those aged 45–64 years, the nationwide ASIRs in 2019 were 35.3 and 31.1 per 100 000 in men and women, respectively. We found an increase of 1.8% in men (95% CI = 0.5; 2.5) and 1.5% in women (95% CI = 0; 2.3) during the period 2003–2019.

**Table 1:** Joinpoint analysis of the age-standardised incidence rate of melanoma (ICD-10 C43) in Hungary in 45–64 years old (2001–2019)

		Men				Women			
Region (NUTS−2)	County (NUTS−3)	Interval	APC	Lower CI	Upper CI	Interval	APC	Lower CI	Upper CI
Pest	Pest	2001–2016	5.6*	3.3	22.3	2001–2003	67.3*	14.3	129.2
		2016–2019	−5.9	−25.6	5.1	2003–2019	2.6	−1.4	4.6
Budapest	Budapest	2001–2003	35.6*	6.6	63.1		3.1*	1.4	4.7
		2003–2019	1.6	−1.2	2.9				
Central Transdanubia	Komárom Esztergom	2001–2017	7.4*	3.0	49.9		−1.0	−4.5	2.5
		2017–2019	−42.0	−65.4	3.4				
	Fejér		3.4	−0.6	7.4		2.9*	0.1	5.7
	Veszprém		0.5	−2.2	3.3		2.0	−1.3	5.3
Western Transdanubia	Győr−Moson−Sopron		−0.6	−4.7	3.7		−1.2	−5.0	2.7
	Vas		0.3	−3.4	4.1	2001–2008	11.8*	2.3	53.9
						2008–2019	−7.1*	−20.5	−2.6
	Zala		−0.11	−3.2	3.1	2001–2007	−9.8*	−29.3	−1.5
						2007–2019	5.7*	2.4	17.0
Southern Transdanubia	Baranya		1.1	−2.7	5.1		3.9	−0.5	8.6
	Somogy	2001–2009	12.0*	2.8	120.6	2001–2003	91.1*	9.7	198.7
		2009−2019	−5.9	−48.3	0.1	2003–2019	1.8	−8.1	5.0
	Tolna		−0.5	−6.0	5.4		4.0	−1.6	9.9
Northern Hungary	Borsod−Abaúj−Zemplén		3.8*	0.8	7.0		3.9*	0.01	8.0
	Heves		3.2*	0.1	6.5		−0.7	−4.4	3.0
	Nógrád		5.1	−0.4	10.7		5.2*	1.3	9.3
Northern Great Plain	Hajdú Bihar	2001–2003	69.6*	5.9	150.8	2001–2004	83.8*	28.9	250.3
		2003–2019	0.7	−11.9	3.5	2004–2019	−4.1*	−9.0	−0.5
	Jász−Nagykun−Szolnok		3.1	−0.3	6.7		−1.2	−5.5	3.2
	Szabolcs−Szatmár−Bereg		3.9*	1.2	6.7		0.8	−2.7	4.4
Southern Great Plain	Bács−Kiskun		1.9	−0.6	4.4		1.5	−2.0	5.1
	Békés		1.4	−1.6	4.5		0.01	−4.0	4.2
	Csongrád−Csanád		0.6	−2.3	3.6	2001–2004	34.4*	2.7	126.3
						2004–2009	−1.1	−27.0	2.3
Hungary		2001–2003	23.9*	7.5	39.9	2001–2003	23.0*	6.0	38.8
		2003–2019	1.8*	0.5	2.5	2003–2019	1.5*	0.03	2.3

*Abbreviations*: *NUTS-2* (Nomenclature for Territorial Units for Statistics Level 2) refers to regions, *NUTS-3* (Nomenclature for Territorial Units for Statistics Level 3) refers to counties, *APC* Annual percent change, *CI * Confidence interval

*Significantly different from zero at α = 0.05

**Table 2:** Joinpoint analysis of the age-standardised incidence rate of melanoma (ICD-10 C43) in Hungary, 65+ years old (2001–2019)

		Men				Women			
Region (NUTS−2)	County (NUTS−3)	Interval	APC	Lower CI	Upper CI	Interval	APC	Lower CI	Upper CI
Pest	Pest		6.0*	4.1	7.8		5.0*	2.8	7.2
Budapest	Budapest		5.1*	3.7	6.6		5.5*	4.0	7.0
Central Transdanubia	Komárom Esztergom		6.9*	2.7	11.3	2001–2008	19.6*	10.8	41.2
						2008–2019	−3.3	−9.8	0.9
	Fejér	2001–2003	110.0*	38.2	186.1	2001–2005	17.5*	4.3	53.0
		2003–2019	5.0*	1.7	7.3	2005–2017	−1.6	−18.1	0.5
						2017–2019	45.3*	6.0	76.7
	Veszprém		5.1*	0.3	10.1	2001–2004	42.1*	5.8	138.4
						2004–2019	−0.4	−14.3	2.7
Western Transdanubia	Győr−Moson−Sopron		7.3*	3.2	11.5	2001–2005	25.1	−1.2	166.4
						2005–2019	−0.25	−46.6	11.3
	Vas		4.0*	0.9	7.2		4.2*	−0.7	9.3
	Zala		2.2	−1.5	6.0	2001–2005	−22.0	−50.7	−1.6
						2005–2013	14.4*	6.5	61.3
						2013–2019	−5.4	−40.7	4.6
Southern Transdanubia	Baranya					2001–2011	−3.2	−27.6	2.5
			6.0*	2.5	9.6	2011–2019	11.9*	3.3	57.9
	Somogy		6.6*	2.4	10.9	2001–2015	6.9*	4.1	28.0
						2015–2019	−11.1	−39.4	3.1
	Tolna		4.8*	0.2	9.5		3.8*	1.5	6.2
Northern Hungary	Borsod−Abaúj−Zemplén		8.4*	6.3	10.5		6.5*	4.2	8.9
	Heves		7.7*	4.6	11.0	2001−2008	19.9*	11.1	42.8
						2008−2019	−2.4	−9.5	1.8
	Nógrád		4.0*	0.2	7.9		5.3*	2.9	7.7
Northern Great Plain	Hajdú Bihar		6.6*	3.0	10.3		3.7*	0.4	6.9
	Jász−Nagykun−Szolnok		6.9*	4.4	9.5		1.7	−1.2	4.6
	Szabolcs−Szatmár−Bereg		4.6*	0.8	8.5		2.5	−1.3	6.3
Southern Great Plain	Bács−Kiskun	2001–2003	99.0*	19.3	231.9	2001–2006	−8.1	−32.8	4.1
		2003–2006	−40.8	−53.5	−16.3	2006–2019	5.8	−11.2	29.7
		2006–2009	47.3*	11.7	86.3				
		2009–2019	3.6	−15.7	9.2				
	Békés		4.6*	0.5	8.8		3.4*	0.5	6.4
	Csongrád−Csanád		3.7*	0.6	6.9		2.7*	0.1	5.4
Hungary		2001–2003	17.2*	9.8	25.3		4.1*	3.1	5.2
		2003–2010	4.1	−0.2	5.1				
		2010–2015	8.2*	6.2	12.6				
		2015–2019	0.4	−4.9	2.8				

*Abbreviations*: *NUTS-2* (Nomenclature for Territorial Units for Statistics Level 2) refers to regions, *NUTS-3* (Nomenclature for Territorial Units for Statistics Level 3) refers to counties, *APC* Annual percent change, *CI* Confidence interval

*Significantly different from zero at α = 0.05

In the county-level analysis, trends were similar across three counties in men (Borsod-Abaúj-Zemplén: APC = 3.8 (95% CI = 0.8, 7.0), Heves: APC = 3.2, 95% CI = 0.1; 6.5), and Szabolcs-Szatmár-Bereg: APC = 3.9 (95% CI = 1.2; 6.7) from 2001 to 2019. In other counties, various intervals were revealed. Typically, the rate of increase was greater in the first interval of the study period, followed by non-significant changes in the trend. In Pest, the APC was 5.6% for 2001–2016 (95% CI = 3.3; 22.3). In Komárom-Esztergom, the upward trend persisted until 2017 (APC = 7.4, 95% CI = 3.0; 49.9). In Somogy, the significant interval was in 2001–2009 (APC = 12.0, 95% CI = 2.8; 120.6).

In women, the geographical pattern resembled the national and male patterns in some counties. In Hajdú-Bihar county, a reduction was observed between 2004 and 2019 (APC = − 4.1, 95% CI = − 9.0; -0,5). In Vas county, an initial increase of 11.8% (95% CI = 2.3; 53.9) from 2001 to 2008 was followed by a decrease (APC = − 7.1, 95% CI = − 20.5; −2.6). In neighbouring Zala, the opposite occurred: after a decrease, an increasing trend followed (APC (2001–2007) = − 9.8, 95% CI = − 29.3; −1.5), APC (2007–2009) = 5.7, 95% CI = 2.4; 17.0). Somogy county showed an increase of 91.1% (95% CI = 9.7; 198.7) until 2009, which was seven times greater than in men. In Budapest, Fejér, Borsod-Abaúj-Zemplén an increasing trend was observed without any joinpoints (Budapest: APC = 3.1, 95% CI = 1.4; 4.7, Fejér: APC = 2.9, 95% CI = 0.1; 5.7), Borsod-Abaúj-Zemplén: APC = 3.9, 95% CI = 0.01; 8.0).

In Hungary, the 65 or older population had an ASIRs of 115.3 and 56.2 per 100 000 in men and women, respectively, in 2019. Significant interval was detected in men from 2010 to 2015 (APC = 8.2, 95% CI = 6.2; 12.6) and in women without any breakpoints (APC = 4.1, 95% CI = 3.1; 5.2).

At the county-level, all incidence changes were significant in the older age group. The greatest changes were detected in Borsod-Abaúj-Zemplén (APC = 8.4, 95% CI = 6.3; 10.5) and Heves (APC = 7.7, 95% CI = 4.6; 11.0). In Fejér, the relevant segment showed an APC of 4.95% (95% CI: 1.7; 7.3) for the period 2003–2019. Apart from Bács-Kiskun, where three joinpoints were found, and the trends have stagnated since 2009, the rest of the counties have shown a consistent linear increase without any breakpoints.

In women, the pattern was more variable. In counties without joinpoints, Borsod-Abaúj-Zemplén had the highest APC (APC = 6.5, 95% CI = 4.2; 8.9), and Budapest (APC = 5.5, 95% CI = 4.0; 7.0). In Komárom-Esztergom, Győr-Moson-Sopron, Veszprém, Zala, Somogy and Heves, the trends stagnated after a rise that occurred at different times.

Age group analysis (shown in Table 3) of women revealed an increase of 9.3% in the last ten years of the study period in the 45–54 year-old population (2nd segment 2011–2019, APC = 9.3 (95% CI = 3.3; 35.6) populations. A variable segmentation pattern was observed in all the other age groups (men and women) except for the last age group (75–84 years old), which showed a steady 6.4% annual increase in men (95% CI = 5.8; 7.1).

**Table 3:** Age-group analysis of age-standardised melanoma (ICD-10 C43) incidence rates in Hungary (2001–2019)

Age group (years)	Men				Women			
	Intervals	APC	Lower CI	Upper CI	Intervals	APC	Lower CI	Upper CI
45−54	2001–2003	17.3*	3.5	31.7	2001–2011	−10.2*	−36.2	−0.3
	2003–2019	3.4	−4.9	5.1	2011–2019	9.3*	3.3	35.6
55−64	2001–2003	27.8*	6.4	50.7	2001–2006	−14.8*	−39	−2.8
	2003–2019	0.8	−1.0	1.7	2006–2015	8.8*	3.9	40.7
					2015–2019	−12.1	−40.7	0.7
65−74	2001–2015	5.4*	4.6	8.1	2001–2005	−20.2	−47.7	0.3
	2015–2019	1.7	−11.7	3.4	2005–2013	14.6*	6.5	62.8
					2013–2019	−8.4	−35.8	1.7
75−84		6.4*	5.8	7.1		3.4	−2.3	9.4

*Abbreviations*: *APC* Annual percent change, *CI* Confidence interval

*Significantly different from zero at α = 0.05

In 2019, ASDR was 3.1 per 100 000 individuals in the 45–64 year-old group and 13.3 per 100 000 individuals in the 65 year-old or older group. ASDR in the 45–64 year-old population decreased in all regions by 2019 (Table 4). In Budapest, a significant decrease (− 13.4%) was found between 2013 and 2019 (95% CI = − 35.1; −6.9). In Central Transdanubia, three segments were detected with a notable decrease in the last interval (APC (2015–2019) = − 18.2;

**Table 4:** Joinpoint analysis of the age-standardised death rate for melanoma (ICD-10 C43) in Hungary (2001-2019)

	45–64 years old				65+ years old			
Region	Interval	APC	Lower CI	Upper CI	Interval	APC	Lower CI	Upper CI
Pest		−0.2	−4.2	4.1		1.9	−0.2	4.0
Budapest	2001–2013	−2.9	−5.5	16.4		−1.6	−3.3	0.3
	2013–2019	−13.4*	−35.1	−6.9				
Central Transdanubia	2001–2003	−30.6*	−44.2	−0.03		0.1	−3.0	3.2
	2003–2015	6.1*	3.0	29.7				
	2015–2019	−18.2*	−39.4	−4.0				
Western Transdanubia	2001–2011	8.7*	1.0	88.6		5.4*	1.8	9.0
	2011–2019	−10.7*	−50.3	−1.3				
Southern Transdanubia		−2.3	−5.2	0.7		1.4	−0.31	3.0
Northern Hungary		−3.4*	−5.6	−1.1		1.0	−1.7	3.7
Northern Great Plain		−1.4	−4.2	1.4		−0.02	−2.4	2.5
Southern Great Plain		−0.7	−4.0	2.8		0.3	−1.6	2.3
Hungary	2001–2013	0.1	0.001	0.1	2001–2007	−0.02	−0.06	0.1
	2013–2016	−0.4	−0.5	0.1	2007–2019	−0.08*	−0.2	−0.06
	2016–2019	0.3	−0.1	0.7				

NUTS-2 regions: Central Transdanubia (Komárom-Esztergom, Fejér, Veszprém), Western Transdanubia (Győr-Moson-Sopron, Vas, Zala), Southern Transdanubia (Baranya, Somogy, Tolna), Northern Hungary (Borsod-Abaúj-Zemplén, Heves, Nógrád), Northern Great Plain (Hajdú-Bihar, Jász-Nagykun-Szolnok, Szabolcs-Szatmár-Bereg), Southern Great Plain (Bács-Kiskun, Békés, Csongrád-Csanád

*Abbreviations:NUTS-2* (Nomenclature for Territorial Units for Statistics Level 2) refers to regions, *NUTS-3* (Nomenclature for Territorial Units for Statistics Level 3) refers to counties, *APC* Annual percent change, *CI* Confidence interval

*Significantly different from zero at α = 0.05

95% CI = − 39.4; −4.0). In Western Transdanubia, two trends were observed: an increase of 8.7% from 2001 to 2011 (95% CI = 1.0; 88.6) and a 10.7% decrease in 2011–2019 (95% CI = − 50.3; −1.3. In the older cohort, the only significant change was observed in Western Transdanubia, where the ASDR increased by 5.4% (95% CI = 1.8; 9.0) between 2001 and 2011.

## Discussion

The recent descriptive study explored the long-term spatial patterns of melanoma incidence and mortality in Hungary. Our goal was threefold. First, to characterise the incidence rates from 2001 to 2019; second, to identify the age group in which the incidence of melanoma increased most rapidly; and third, to determine regional patterns of mortality trends over the study periods.

County-level analysis indicated that Budapest, Pest, Nógrád, Borsod-Abaúj-Zemplén, Fejér, Komárom-Esztergom, Győr-Moson-Sopron, Zala, Heves, Csongrád-Csanád and Hajdú-Bihar counties experienced the most substantial increase in melanoma cases, irrespective of sex and age group. Our finding in Hajdú-Bihar corroborates the study by Janka et al., who reported a continuously increasing incidence of invasive melanoma in males above the age of 60 years based on the hospital registry at the University of Debrecen, which represented the Northern Great Plain (Hajdú-Bihar, Jász-Nagykun-Szolnok and Szabolcs-Szatmár-Bereg) [7]. In contrast to our results, Janka et al. [26] found significant increases only in Győr-Moson-Sopron county [26]. However, the aforementioned study applied different database, shorter time interval, and compared to the data of HNCR, the published case numbers were overestimated.

A recent study by Wéber et al. [27] found that melanoma is among the cancers for which the largest regional (county-level) ASIRs differences exist in Hungary. The authors noted that melanoma incidence increased over the period 2005–2019 in many counties: In Borsod-Abaúj-Zemplén, Zala, Bács-Kiskun, Baranya and Pest showed the highest changes in 2015–2019 vs. 2005–2009. Their study did not include subgroup analyses, which may explain the differences observed compared with our study [27].

Although our research did not extend to the analysis of socioeconomic and environmental factors, we attempt to discuss the regional variations and the potential socioeconomic causes of the observed spatio-temporal trends.

Counties of lower socioeconomic status (SES), such as Somogy, Borsod-Abaúj-Zemplén, Nógrád and Heves, presented the highest annual changes in ASIRs [28, 29]. Our spatial findings are unexpected because many studies have demonstrated that SES inequalities can act in the opposite direction to other cancer types. Janka et al. [26] reported that single-person households, lower educational levels and unemployment were negatively correlated with the incidence of melanoma [26]. Overall, the general position is that melanoma is more common in high-income countries [30] or at least associated with early-stage melanoma diagnosis [31]. People from affluent areas have a better prognosis for many forms of cancer than those from deprived neighbourhoods and dilapidated housing [32].

Pest county, a suburban area in the proximity of Budapest with 1.3 million inhabitants (2023 data), and Budapest were still frontrunners in the increased melanoma trends for 65 year-old or older men and women [33]. Budapest and Pest are by far the best-performing economic areas in the country. In addition, Budapest comprises 23 districts with significant socio-economic variations [34]. One possible explanation for the increased incidence in Budapest and Pest compared with the rest of the country could be that early screening campaigns are more successful in Budapest and its conurbation area. In addition, private outpatient capacity is the highest in this region, which suggests a greater utilization of dermatology services [35]. Whether this is a case of overdetection, as argued by Adamson et al. [36], requires further discussion [36]. However, it is also conceivable that lifestyle-associated factors, including the financial ability to take annual holidays, may also lead to a greater risk of melanoma. The stage at diagnosis and carcinoma in situ status must be further evaluated to confirm this hypothesis.

In the older population, the incidence rate increased among women until 2015 but then stagnated. For men, the increase was uninterrupted. Janka et al. [26] demonstrated that the age-standardised incidence rate stagnated between 2013 and in 2017 in the entire country, which supports our findings [26]. From a global perspective, these findings are consistent with those reported by Berk-Krauss et al. (2024) and Schuurman et al. (2019) [37, 38]. With respect to sex differences in the incidence rate of older populations studies from North America, Western Europe, and Australia similarly reported substantially higher incidence rates in older men than in older women, typically ranging from 1.5- to 2-fold differences [39–41]. In our cohort, older men had an incidence rate nearly twice that of women in the same age groups. The rates were similar in younger individuals. However, lowering the age cut-off would likely have resulted in a higher incidence among women than among men.

Our second goal, the age band analysis, proved to be the most meaningful. In the group of 45– to 54 year-old women, the increase was most prominent in the last 10 years of the study period. Explanations for the different ages at onset include sex-specific behavioural differences, such as differences in clothing habits, sun-seeking travel, winter holidays, and sex-specific anatomic sites [42–44]. The growing popularity of sunbeds, which is classified as carcinogenic by the International Agency for Research on Cancer, could also be implicated in the increased incidence of melanoma in this age group of women [45]. A 2004 study that assessed sunbed users’ motivation in Hungary revealed that 64% of users were women; however, the average age was similar for both sexes (< 30 years old) [46]. Sunbed use has been associated with early-onset melanoma; therefore, it is plausible that sunbed use caused the increase in this cohort [47]. Overall, the literature is mixed on whether the age at diagnosis has shifted to a younger age group. Our observations could indicate that either prevention campaigns and sunscreen advertisements are ineffective or could be a sign of successful early detection programs [48, 49].

Our third purpose was to study long-term regional mortality trends. We showed that mortality increased only in the Western Transdanubia in 65 year-old or older individuals; however, this finding may be random due to the low case numbers. Nevertheless, we suggest that health inequities may contribute to geographical differences in mortality trends.

Hungary has faced the problem of medical emigration for decades. The number of doctors who emigrated or left the medical profession was significant (ca. 6%) between 2009 and 2017 [50]. Central Hungary has almost twice as many doctors per capita as Northern Hungary, resulting in doctor shortages in economically disadvantaged and rural areas [51, 52]. Unfilled GP practices are typical for the entire country but are more pronounced in deprived areas [53]. Furthermore, the number of GPs was lowest in northeastern and southwestern counties and Southern Transdanubia [54]. In 2021, clinical oncology was listed as a shortage profession in Bács-Kiskun, Békés, Tolna and Vas; dermatology in Budapest; and Komárom-Esztergom and pathology in Budapest, Heves, Veszprém and Zala [55]. Additionally, towns near the Austrian border attract doctors to work in medical tourism, potentially creating shortages in basic care in rural areas. This disproportionate distribution of doctors may lead to discovery of melanoma at later stages and, consequently, higher mortality rates, for example, in Western Transdanubia.

The distribution of healthcare services was also not balanced. In 2015, Southern Transdanubia had long waiting lists, whereas Western Transdanubia had lower outpatient capacity than did Budapest and Pest counties [56].

The mortality rate for 45–64 years old individuals decreased significantly in Budapest and Central Transdanubia in the second half of the study period, this finding is consistent with the results of Wéber et al. (2024) [27]. This could suggest early detection and prompt oncologic care [6].

The contribution of this study lies in its comprehensive regional and age-specific trend analysis of melanoma incidence and mortality across nearly two decades (2001–2019), using age-standardised rates at the NUTS-3 level and Joinpoint regression to identify statistically significant temporal changes, which allows the detection of localised and age-dependent shifts in melanoma trends that national-level analyses may overlook.

The main limitation of our study is the patients’ permanent residence in HNCR and HCSO may differ from their habitual residence, which could have affected our arguments on SES. Wide CIs, which can arise from small sample sizes (low event counts in certain counties) or high interannual variability, must be interpreted cautiously regardless of statistical significance, however these could indicate a change in tendency.

We could also not verify the proposition of Frangos et al. [57] that the increased incidence rates in younger adults are due to the upstaging of thin melanomas or the overbiopsy of the population [57]. That work questioned the global rise in melanoma incidence and explained the apparent rise in incidence with the changing of pathologists’ practice on upstaging of gray-zone “dysplastic nevus” diagnosis to thin cutaneous melanomas.

Lastly, limitations could arise from data quality issues associated with both incidence and mortality data. Miscoding and missing biopsy results could contribute to the feasibility of incidence data in HNCR. Furthermore, postmortem diagnoses are not always included in HNCR, the literature called this phenomenon as “Death Certificate Only” cases, however this constitutes a negligible proportion of the cases (0.1% in 2018). In the same year, about 55% of melanoma cases were morphologically verified [16]. The primary cause of death is usually good quality and is verified by a medical examiner [58].

## Conclusions

By extending county-level analysis to 20 years, our research adds to the body of evidence in melanoma research in Hungary. Our results revealed a modest increase in the incidence of melanoma between 2001 and 2019 in older cohorts. The results indicated decreasing incidence trends in Vas and Hajdú-Bihar in the 45–64 year-olds. Perhaps the most remarkable spatial finding is the dynamic change observed in some counties, for example, in Borsod-Abaúj-Zemplén and Nógrád, which have poor socioeconomic conditions. While previous studies generally report a linear relationship between socioeconomic status and melanoma incidence rates, our findings suggest that this association may differ in the Hungarian context. Our main message for policy-makers is that in Hungary, melanoma is most frequently observed among women aged 45 to 54 years of age. This finding highlights the urgency of efficient prevention and early detection campaigns.

Melanoma mortality in most regions stagnated or decreased, except for Western Transdanubia among the population above 65 years of age. However, this finding may be random due to the low case numbers.

We demonstrated geographical differences in melanoma epidemiology in Hungary at the county level. Further study at the district level (Local administrative unit-1 (LAU-1, formerly NUTS-4)) is warranted.

## Supplementary Information

Supplementary Material 1

Supplementary Material 2

## Data Availability

The data that support the findings of this study are openly available on https://stat.nrr.hu/, which is maintained by the Hungarian Cancer Registry. Cancer mortality data was obtained through official data sharing between public institutions. This paper has been prepared using the tabular data set (Population per district 5 years sex 2001-2019) compiled by the Central Statistical Office in response to a specific request. The calculations and the conclusions drawn from these data set are the authors’ intellectual property.

## Abbreviations

APC: Annual Percent Change
ASIR: Age-standardised incidence rates
ASDR: Age-standardised death rates
CI: Confidence interval
HCSO: Hungarian Central Statistical Office
HNCR: Hungarian National Cancer Registry
NCPHP: National Center for Public Health and Pharmacy
NHIF: National Health Insurance Fund
NUTS: Nomenclature of Territorial Units for Statistics
SES: Socioeconomic status
